# Recognition of bird species with birdsong records using machine learning methods

**DOI:** 10.1371/journal.pone.0297988

**Published:** 2024-02-23

**Authors:** Yi Tang, Chenshu Liu, Xiang Yuan

**Affiliations:** 1 School of Emergency Management, Institute of Disaster Prevention, Sanhe, China; 2 Hebei Key Laboratory of Resource and Environmental Disaster Mechanism and Risk Monitoring, Sanhe, China; 3 Samueli School of Engineering, Department of Bioengineering, University of California Los Angeles, Los Angeles, CA, United States of America; 4 School of Life Science, Liaoning University, Shenyang, China; Universidad Adolfo Ibanez, CHILE

## Abstract

The recognition of bird species through the analysis of their vocalizations is a crucial aspect of wildlife conservation and biodiversity monitoring. In this study, the acoustic features of *Certhia americana*, *Certhia brachydactyla*, and *Certhia familiaris* were calculated including the Acoustic complexity index (ACI), Acoustic diversity index (ADI), Acoustic evenness index (AEI), Bioacoustic index (BI), Median of the amplitude envelop (MA), and Normalized Difference Soundscape Index (NDSI). Three machine learning models, Random Forest (RF), Support Vector Machine (SVM), and Extreme Gradient Boosting (XGBoost), were constructed. The results showed that the XGBoost model had the best performance among the three models, with the highest accuracy (0.8365) and the highest AUC (0.8871). This suggests that XGBoost is an effective tool for bird species recognition based on acoustic indices. The study provides a new approach to bird species recognition that utilizes sound data and acoustic characteristics.

## 1. Introduction

The recognition of bird species is essential for biodiversity conservation and wildlife management [1, 2]. Birds play critical roles in ecosystems and serve as indicators of environmental health, and the study of bird species provides valuable information for further research and conservation efforts [3]. By monitoring bird populations and identifying potential threats to their survival, conservationists can make informed decisions about land use and habitat preservation [4, 5].

Birdsong is a crucial aspect of bird species recognition and plays a significant role in the study of birds [6]. Its unique and distinctive qualities make it an essential tool for accurately assessing bird populations, monitoring changes in distribution and behavior, and informing conservation efforts [7, 8]. The ability to recognize birds by their song allows ornithologists and conservationists to determine the presence of different bird species in specific areas and track changes in populations over time [9]. This information can then be used to inform conservation efforts and ensure the preservation of bird populations.

Correct identification of birds is important for understanding and protecting birds, protecting biodiversity, and realizing sustainable development [10]. Biodiversity monitoring provides essential information for conservation actions to mitigate the threat and loss of species, especially in the context of climate change [11].

The analysis of birdsong is a valuable tool for bird species recognition and has been used for decades in the field of ornithology [6]. Acoustic analysis involves identifying birds based on their vocalizations, such as songs, calls, and other sounds [12]. This method is particularly useful for species identification in areas where visual observation is difficult, such as dense forests or at night. In recent years, advances in technology have made it possible to use computer-based analysis of birdsong to automatically classify bird species, providing accurate and efficient results [13]. In addition, the analysis of birdsong provides important insights into bird behavior and physiology, as different bird species, or even individual birds, emit different songs in response to different environmental stimuli [14]. The study of birdsong therefore plays a critical role in bird species recognition and the understanding of bird behavior and ecology.

Traditional Acoustic Analysis for bird species recognition involves manually examining various acoustic features, such as frequency, tempo, and duration, to distinguish between different bird species [15, 16]. Despite producing valuable results, this method has limitations in terms of efficiency and accuracy, and can result in errors and difficulties in conducting research. High costs and low spatial and temporal coverage are some of the challenges faced by this method. Thus, it is important to explore alternative methods for bird species identification.

Recently, machine learning methods for bird species recognition have emerged as a promising alternative to traditional acoustic analysis [17]. These methods harness the capabilities of machine learning, a subset of techniques within the broader field of artificial intelligence, to automatically extract and analyze acoustic features from birdsong recordings. For example, researchers have used deep neural networks, a category of machine learning, to classify bird species based on their songs and have achieved high accuracy in their results [18].

The use of public bird song records and machine learning methods for recognition has become an increasingly popular approach in bird species identification [19]. This involves collecting and storing large amounts of bird song data in public databases, which can be used to train machine learning algorithms to recognize different bird species based on their unique vocalizations. The algorithms can then be applied to new recordings to identify unknown bird species. This approach has several advantages over traditional methods, including the ability to process large amounts of data quickly and accurately, the ability to identify birds in real-time, and the ability to handle complex variations in bird vocalizations. Some of the most commonly used machine learning methods for bird species recognition include deep neural networks, support vector machines, and random forest [20, 21]. However, the success of this approach also depends on the quality and quantity of the bird song data used for training, and on the effectiveness of the machine learning algorithms used for recognition.

The comparison of machine learning methods for bird species recognition is crucial. The choice of method affects the accuracy and efficiency of bird species recognition systems, and comparing the performance of different algorithms such as random forest, support vector machines, and neural networks can inform the selection of the best method and guide the development of new ones. This research can contribute to the advancement of the field and lead to more effective and efficient recognition systems for use in wildlife conservation and biodiversity monitoring [22].

The objective of this study is to identify bird species using their acoustic characteristics obtained from birdsong recordings. To fulfill this purpose, the acoustic features were initially extracted, and then partitioned into training and testing datasets. Based on datasets, machine learning models were developed and their performance was compared to evaluate the differences among methods here.

## 2. Methods and materials

### 2.1 Birdsong classification network

**Fig 1:**
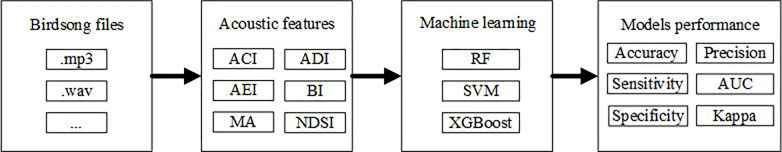
Data flow diagram for recognition of bird species with birdsong records.

In the process of classifying birdsong, we adopted a comprehensive methodological framework as depicted in Fig 1. Initially, we collected diverse vocalizations from different bird species, ensuring a varied dataset from multiple environments. Subsequently, we extracted key acoustic features such as the Acoustic Complexity Index (ACI), Acoustic Diversity Index (ADI), Acoustic Evenness Index (AEI), Bioacoustic Index (BI), Median Amplitude (MA), and Normalized Difference Soundscape Index (NDSI), which are instrumental in characterizing the unique vocal patterns of each bird species. Following the preprocessing, we developed and trained three machine learning models: Random Forest (RF), Support Vector Machine (SVM), and Extreme Gradient Boosting (XGBoost), each fine-tuned for optimal performance using the calculated features. The models were then rigorously evaluated using a range of metrics including accuracy, Area Under Curve (AUC), kappa, precision, sensitivity, and specificity, providing a comprehensive assessment of their classification capabilities. To effectively address potential data imbalance within our dataset, we adopted a One-vs-All strategy. This approach entailed individually computing key indices, such as the AUC, for each bird species. Here, we treated each species in turn as the positive class, while grouping all other species as the negative class. We calculated these indices for every species and then averaged them to obtain an overarching evaluation metric. This methodology played a crucial role in mitigating the impacts of data imbalance, thereby enhancing the robustness of our analysis.

### 2.2 Dataset sources

The bird song audio public data used in this study was obtained from the Xeno-canto website (https://www.xeno-canto.org), which is a globally recognized platform for sharing bird vocalizations. The website is renowned for its extensive collection of bird audio recordings from various environments, contributed by users worldwide [19]. Here, we initially explored high-quality audio recordings of the Certhia genus and identified 975 audio files spanning 10 different species. Upon preliminary review, we observed that some species had limited audio files available, such as *Certhia manipurensis* (n = 5, where "n" indicates the number of audio files), *Certhia nipalensis* (n = 2), and *Certhia tianquanensis* (n = 3). To ensure a robust analysis, we decided to focus our study on three species with a more substantial number of audio files, namely *Certhia americana* (n = 101), *Certhia brachydactyla* (n = 393), and *Certhia familiaris* (n = 375).

### 2.3 Acoustic indices

The acoustic characteristics of birdsongs were calculated using various acoustic indices, including Acoustic complexity index (ACI), Acoustic diversity index(ADI), Acoustic evenness index(AEI), Bioacoustic index (BI), Median of the amplitude envelop (MA), and Normalized Difference Soundscape Index(NDSI). The complete meaning of each acoustic index is shown in Table 1.

**Table 1:** The meaning of acoustic indices in this study.

Indices	Equations	Meaning
ACI	$ACI=\Sigma_{i-1}^{N-1}\Sigma_{j-1}^{M}|x_{i,j}-x_{i+l,j}|$, where *x*_*i*,*j*_ represents the amplitude at time *i* and frequency bin *j*	a metric that quantifies the variety of sounds within a soundscape
ADI	$ADI=-\Sigma P_{i}log(P_{i})$ where *P*_*i*_ is the proportion of the total energy in the *i*th frequency bin	a measure of the variability of sounds in a soundscape
AEI	$AEI=\frac{exp(ADI)}{S}$ where *S* is the total number of frequency bins.	a measure of how evenly the soundscape is divided between different sound types
BI	$BI=\frac{E_{N}-E_{S}}{E_{N}+E_{S}}$ where *E*_*N*_ is the energy in the noise band and *E*_*S*_ is the energy in the signal band.	a measure of the overall acoustic quality of a soundscape, taking into account the sound pressure level, frequency range, and duration of the sounds
MA		a measure of the overall loudness of a soundscape
NDSI	$NDSI=\frac{BI-AEI}{BI+AEI}$	a measure of the difference between a soundscape and a baseline soundscape, allowing for comparison between different soundscapes

### 2.4 Machine learning models

There machine learning methods considered in this study include Random Forest (RF), Support Vector Machine (SVM) and Extreme Gradient Boosting (XGBOOST). Random Forest is a versatile technique applicable to both regression and classification problems and has gained prominence in ecology and environmental science for modeling complex relationships between environmental variables and ecological outcomes.The construction of the trees continues until a predetermined stopping criterion, such as a minimum number of samples in each group or a maximum tree depth, is satisfied. It operatesby constructing multiple decision trees, where each tree is built using a random subset of variables to partition the data into smaller groups.The construction of the trees continues until a predetermined stopping criterion, such as a minimum number of samples in each group or a maximum tree depth, is satisfied.

SVM is a supervised machine learning algorithm that is used for classification and regression problems in ecology and environmental science. The algorithm works by mapping the data into a higher dimensional space, finding a linear boundary that separates the data into different classes or predicts continuous values, and making predictions based on the position of new data points relative to the boundary. The boundary is defined by a small number of key data points, called support vectors, which have the greatest impact on the decision boundary. SVM is flexible and powerful, able to handle non-linearly separable data through the use of kernel functions.

XGBoost is a gradient boosting framework that has garnered attention for its ability to model non-linear relationships and intricate interactions in the data. While XGBoost inherently uses tree-based learners which can capture non-linear patterns, its true strength lies in its ability to employ regularization techniques to prevent overfitting. XGBoost employs L1 (Lasso) and L2 (Ridge) regularization to prevent overfitting and allows for early stopping based on validation set performance. Though designed for boosted tree methods, which can be parallelized to expedite computations, it’s crucial to recognize that parallelization is a computational technique, not an inherent feature. While XGBoost has been effective in several ecological and environmental applications, its efficiency and accuracy are problem-dependent.

### 2.5 Experimental scheme

The entire dataset was split into two parts, with 70% of the data designated as the training set and the remaining 30% as the test set. To fine-tune the parameters of the machine learning models, grid search was conducted, utilizing a 10-fold cross-validation method to assess model performance. For RF, we adjusted the mtry parameter with values ranging from 1 to 4. For SVM, we experimented with C and sigma parameters set at 0.1, 1, and 10. Lastly, for XGBoost, we fine-tuned several parameters including max_depth, eta, gamma, colsample_bytree, min_child_weight, and subsample using a predefined grid.

The evaluation of the models was conducted using several performance metrics such as accuracy, Area Under the Receiver Operating Characteristic Curve (AUC), Kappa, precision, sensitivity, and specificity. The choice of these metrics ensures a comprehensive assessment of the models across different aspects. Specifically, Kappa, which ranges from 0 to 1, evaluates the accuracy of a classifier beyond random chance. AUC, on the other hand, is obtained by plotting the True Positive Rate (TPR) against the False Positive Rate (FPR) for all possible thresholds. TPR represents the proportion of positive samples correctly classified as positive, while FPR represents the proportion of negative samples wrongly classified as positive. Given the multi-class nature of our problem, AUC was computed using a one-vs-all approach for each category. This method involves considering a specific category as the positive class and grouping the rest into a single negative set. The performance metrics were calculated for each category separately to identify potential variations in the models’ effectiveness across different categories. Subsequently, the mean of these metrics, computed per category, was presented to provide an overall assessment of the model’s performance. The equations for accuracy, precision, sensitivity, and specificity are provided here.

Accuracy = TP+TN / (TP + TN+ FP+FN)
Precision = TP / (TP + FP)
Sensitivity = TP/ (TP + FN)
Specificity = TN/ (TN+ FP)

Where True Positive (TP) is the number of cases where the model correctly predicted a positive outcome. False Positive (FP) is the number of cases where the model predicted a positive outcome, but the actual result was negative. True Negative (TN) is number of cases where the model correctly predicted a negative outcome. False Negative (FN) is the number of cases where the model predicted a negative outcome, but the actual result was positive.

The calculation of acoustic indices was carried out using the packages tuneR, seewave, and soundecology in R programming language [23–25]. The machine learning models were built and evaluated using the caret package in R [26, 27].

## 3. Results

### 3.1 Descriptive statistics

**Fig 2:**
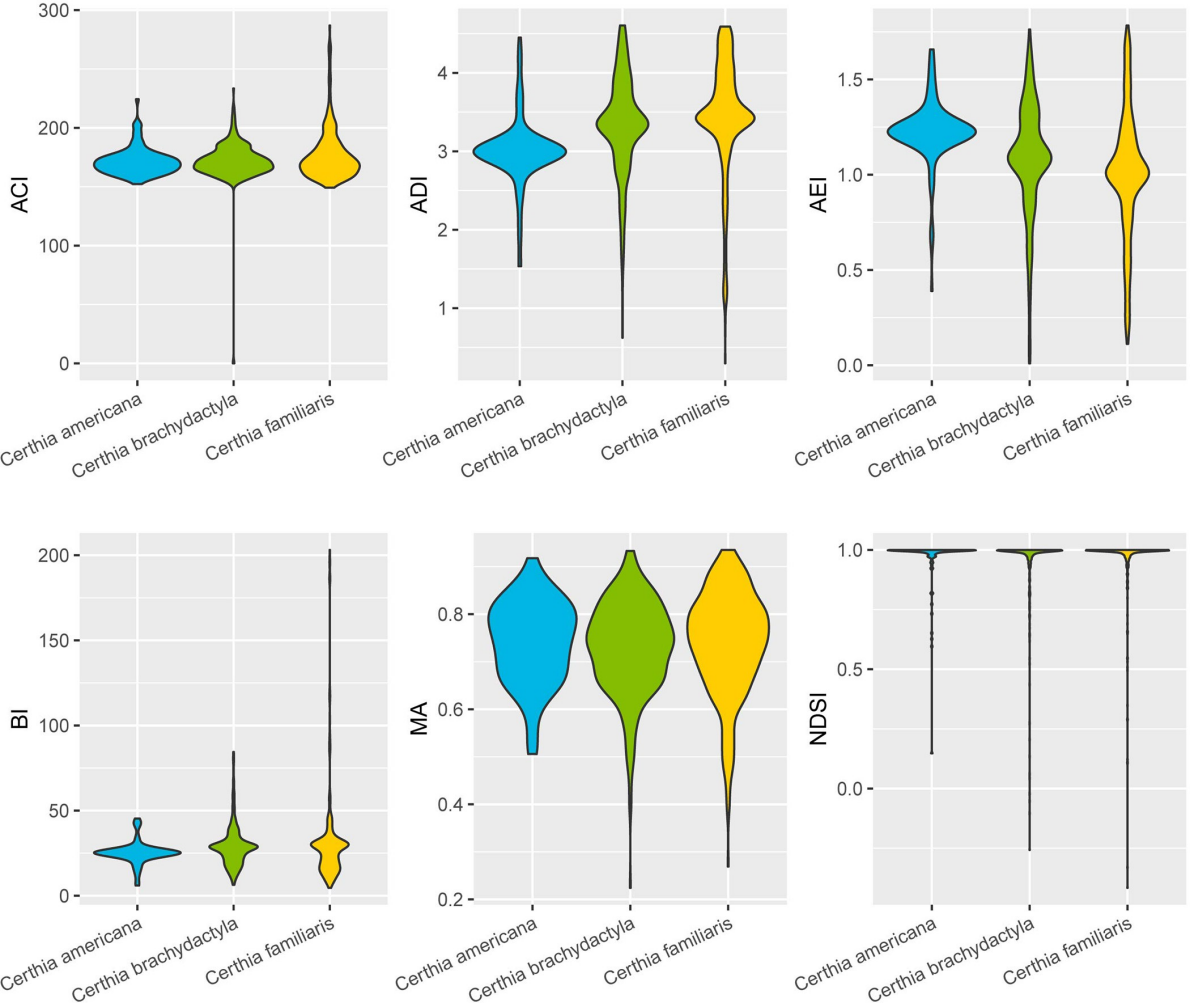
The violin plots of acoustic indices grouped by species.

These acoustic indices serve as predictors to classify the bird species in this study. ACI ranges from a minimum of 0 to a maximum of 287, with a median value of 170.8. ADI ranges from a minimum of 0.2961 to a maximum of 4.6051, with a median value of 3.3812. AEI ranges from a minimum of 0.009 to a maximum of 1.7837, with a median value of 1.0821. BI ranges from a minimum of 4.61 to a maximum of 203.15, with a median value of 27.21. MA ranges from a minimum of 0.2243 to a maximum of 0.9352, with a median value of 0.7447. NDSI ranges from a minimum of -0.4158 to a maximum of 1.0, with a median value of 0.9970. The statistical results grouped by species are shown in Fig 2.

### 3.2 Models comparison

The performance of the three machine learning algorithms used in this study is depicted in Table 2. The table shows the accuracy, AUC, Kappa, precision, sensitivity and specificity for each algorithm. XGBoost algorithm has the highest accuracy (0.8365) and AUC (0.8871) among the three algorithms. The next highest performance was shown by RF, with an accuracy of 0.8314 and AUC of 0.8842, followed by SVM (0.7778, 0.8130). The Kappa values for all algorithms indicate moderate agreement between the predicted and actual class labels, with values ranging from 0.4818 for SVM to 0.6224 for XGBoost. All algorithms have moderate specificity values, with XGBoost having the highest value of 0.8532, followed by RF (0.8509) and SVM (0.8031). The sensitivity values range from 0.6506 for SVM to 0.7331 for XGBoost. In terms of precision, XGBoost has the highest value of 0.8579, followed by RF(0.8509) and SVM (0.8031).

**Table 2:** The performace of machine learning models (Mean±SD).

Models	Accuracy	AUC	Kappa	Precision	Sensitivity	Specificity
RF	0.8314±0.1037	0.8842±0.0496	0.6018±0.1111	0.8545±0.0896	0.8509±0.1250	0.7269±0.0569
SVM	0.7778±0.1437	0.8130±0.1006	0.4818±0.1708	0.8060±0.1271	0.8031±0.1597	0.6506±0.0496
XGBoost	0.8365±0.1090	0.8871±0.0497	0.6224±0.1379	0.8579±0.0880	0.8532±0.1284	0.7331±0.0576

## 4. Discussion

### 4.1 Acoustic indices

The descriptive statistics of the acoustic indices provide valuable insights into the characteristics of different bird species’ vocalizations. For instance, the ACI captures the temporal heterogeneity in sound intensity, which can be indicative of the intricacy of bird calls. We observed a wide range of ACI values across species, suggesting varied complexity in their vocalizations.

Similarly, the ADI, which quantifies the diversity of frequency distribution, showed substantial variation among species. Such differences could potentially be related to the habitat and communication behaviors of these species. The BI, on the other hand, sheds light on the overall acoustic activity in the environment, which can be particularly relevant for understanding the ecological significance of the vocalizations.

By closely examining the distribution and median values of these indices, as illustrated in Fig 2, we gain a more nuanced understanding of the species-specific acoustic behaviors. The violin plots reveal not only the central tendency but also the dispersion in the data, which is crucial for appreciating the heterogeneity in bird vocalizations. Understanding these descriptive statistics is imperative as it lays the foundation for interpreting the results of our classification models. The variability and nuances in these acoustic indices underscore the need for sophisticated machine learning models capable of discerning the subtle differences in vocalization patterns among bird species.

### 4.2 Machine learning models

According to this study, XGBoost model has the best performance among the three models, with the highest accuracy and the highest AUC. It suggests that XGBoost is an effective tool to recognize bird species based on acoustic indices. One advantage of XGBoost over random forest (RF) and support vector machine (SVM) methods is that it has a higher accuracy and AUC. This means that XGBoost is better at accurately recognizing bird species based on acoustic indices. Additionally, XGBoost also has a higher sensitivity value compared to SVM, meaning it is better at detecting positive instances in the data. In terms of precision, XGBoost has a similar value to RF, meaning it is equally good at correctly identifying positive instances and reducing false positive cases. These results suggest that XGBoost is a more effective and efficient tool than RF and SVM for bird species recognition based on acoustic indices.

XGBoost has proven to be a powerful machine learning tool in acoustic research, as demonstrated by its use in bird song classification with Convolutional Neural Networks [28] and the automated identification of frog species in environmental sound recordings [29]. This is because the XGBoost algorithm is particularly well-suited for technical scenarios with complex, non-linear relationships between input features and output targets, making it ideal for recognizing bird species based on acoustic indices which may have complex relationships between spectral and temporal features of bird calls and the species producing them [30, 31].

While it is generally recognized that XGBoost might not be the optimal choice for scenarios with very few training examples or highly imbalanced datasets, in our study, the decision to use XGBoost was based on the comprehensive nature of the dataset for each of the three bird species selected. Despite the limited number of species, the volume of recordings for *Certhia americana* (101), *Certhia brachydactyla* (393), and *Certhia familiaris* (375) provided a substantial dataset, which mitigates concerns regarding small sample sizes. Additionally, the variance in acoustic features across these species offered a sufficiently diverse and representative sample, making XGBoost a suitable model for our analysis. However, we acknowledge that in cases where datasets are extremely limited in sample size or highly imbalanced, other algorithms such as RF or SVM might be more fitting. Also, the computational intensity and complexity of XGBoost should be considered, especially when simpler models might suffice. Therefore, the selection of XGBoost in our study was a measured decision, taking into account the specific characteristics of our dataset. In this study, bird songs are extracted to obtain song features and bird species recognition is performed using machine learning methods. The results indicate that it is possible to perform bird species recognition from the perspective of extracting acoustic features. With the introduction of more machine learning models, the recognition accuracy is expected to continue to improve.

Automatic bird species recognition using machine learning methods is a interesting topic. Previous studies mainly transformed bird vocalizations into images and used convolutional neural networks for bird recognition [18]. There have also been studies on bird classification using sound, such as using Mel-spectrogram coefficients to classify sound [13]. Additionally, audio recordings of environmental sounds have been explored using machine learning methods [32]. However, these studies did not fully utilize sound feature indicators. A recent study has used sound features to study the evolution of bird songs [33], but it did not involve bird recognition. Our study proposes that bird species can be recognized based on their complex acoustic features. This method is different from the traditional approach of transforming bird vocalizations into images for recognition and instead focuses on analyzing sound data and using acoustic features for bird recognition.

### 4.3 Limitation

The study of bird recognition through acoustic features presents both opportunities and challenges. While utilizing sound data and acoustic characteristics to identify bird species holds a wide range of potential, as it offers a departure from traditional methods that involve transforming bird songs into images, it also presents difficulties in extracting and utilizing complex acoustic features of birds. Additionally, the accuracy of the recognition method is partially dependent on the quality and size of the data set, as well as the metrics used. Despite these challenges, the potential benefits of this approach make it a promising avenue for future research in the field of bird recognition, with the aim of gaining new insights into bird behavior and distribution.

In this study, several acoustic indices were utilized to represent the bird vocalization features and to distinguish bird species. These indices effectively capture the characteristics of bird vocalizations. Compared to the previous study, where dissimilarity spaces were used to describe bird vocalization features and classify bird vocalizations [34], the multiple indices used in this study provide a more comprehensive representation of bird vocalization characteristics. It is worth noting that with the enrichment of acoustic indices, the improvement of acoustic acquisition systems, and the enrichment of public databases, there will be more indices and more comprehensive data available for selection in the future.

While the current study provides promising results, it is important to note that the dataset used includes vocalizations from only three species, serving as a preliminary exploration. We acknowledge that validating the efficacy of our method on larger datasets encompassing a broader range of species and vocalization types would be a critical next step. Future studies could extend this work by applying the proposed methodology to larger and more diverse datasets, which would provide a more comprehensive evaluation of the model’s performance and robustness.

## 5 Conclusion

The acoustic features of three bird species, *Certhia americana*, *Certhia brachydactyla*, and *Certhia familiaris*, including ACI, ADI, AEI, BI, MA, and NDSI, were calculated. Three machine learning models, Random Forest (RF), Support Vector Machine (SVM), and Extreme Gradient Boosting (XGBoost), were developed. The results revealed that the XGBoost model had the best performance with the highest accuracy (0.7510) and the highest AUC (0.7222) compared to the other two models, demonstrating the effectiveness of XGBoost in recognizing bird species based on acoustic indices.

This study proposes a method for bird species recognition by analyzing complex acoustic indices from publicly available bird vocalization data and combining it with machine learning models. The study provides a new approach to bird species recognition that utilizes sound data and acoustic characteristics, as opposed to traditional methods that involve transforming bird songs into images. This approach has the potential to significantly enhance our understanding of bird behavior and distribution by providing new insights through its unique analysis of complex acoustic features. Furthermore, the combination of acoustic indices analysis and machine learning models could lead to improved accuracy and efficiency in bird species recognition.

This study presents a novel methodology for bird species recognition, utilizing a direct analysis of complex acoustic indices derived from bird vocalizations. The application of machine learning models, particularly the XGBoost, has been demonstrated to be highly effective in classifying bird species, outperforming traditional methods. Our approach not only offers an innovative perspective but also showcases the potential of machine learning in revolutionizing ecological and environmental studies.

Moreover, the practical implications of this research are profound. The proposed method can enhance the accuracy and efficiency of bird species recognition systems, thereby contributing to improved biodiversity monitoring and conservation efforts. By focusing on sound data and acoustic characteristics, our study lays a robust foundation for future research in this domain and underlines the versatility of machine learning in ecological applications.
